# Understanding research practices and methodological choices when applying the Person-Based Approach to health intervention development: A qualitative interview study

**DOI:** 10.1136/bmjopen-2025-112780

**Published:** 2026-10-07

**Authors:** Lydia Holt, Sarah Denford, Lucy Yardley

**Affiliations:** 1 NIHR Health Protection Research Unit in Evaluation and Behavioural Science, University of Bristol, Bristol, UK; 2 Faculty of Health and Life Sciences, School of Psychological Science, University of Bristol, Bristol, UK; 3 Faculty of Environmental and Life Sciences, School of Psychology, University of Southampton, Southampton, UK

**Keywords:** QUALITATIVE RESEARCH, Patient Participation, Psychosocial Intervention, Research Design, Methods, Behavior

## Abstract

**Abstract:**

**Objective:**

This study explores why researchers selected the Person-Based Approach (PBA) to intervention development for their studies and their perceptions of the strengths and limitations of using the approach in practice.

**Design:**

This is a qualitative interview study, which uses an Interpretive Description approach to thematically analyse data.

**Setting:**

The participants were based at academic institutions across the UK, Europe and North America.

**Participants:**

We interviewed 16 health researchers at different academic career stages, including clinicians, psychologists and social scientists.

**Results:**

Three main themes were chosen to characterise and describe the data: *Navigation* (the PBA provided guidance throughout key development phases, and a clear audit-trail of research decisions); *Flexibility* (the PBA enabled study teams to make resource-responsive adaptations to their research methods, while maintaining the core principle of user focus) and *Integration* (the PBA facilitated the synthesis of diverse evidence sources and the combination of different development methodologies/frameworks).

**Conclusions:**

Using the PBA gave researchers the confidence to adapt their research activities, particularly patient and public involvement and qualitative methods, to better reach target users. They selected the PBA not purely on instrumental grounds, but because it aligned with their professional values and commitment to user-centred practice. The PBA also facilitated the integration of multiple evidence sources and development frameworks. Understanding whether these diverse methodological configurations lead to successful outcomes will require evaluation approaches that can accommodate complexity and pluralism, alongside transparent reporting of the choices made.

**STRENGTHS AND LIMITATIONS OF THIS STUDY**

- Participants had different disciplinary backgrounds, including academic clinicians as well as social and behavioural scientists.
- The analytical approach used, Interpretive Description, was designed specifically for health researchers seeking practice-relevant insights in qualitative data.
- Participants who responded to recruitment may have done so due to positive experiences when using the Person-Based Approach (PBA), which introduces selection bias to the study.
- The sample was predominantly UK based, which may limit transferability of our findings to other research settings.

## Background

### Introduction

 Health behaviour change interventions are complex, targeted at the intersection of individual and societal determinants of health.^1^ They are implemented across diverse community, digital and clinical healthcare settings, and researchers must account for the varied influences and perspectives that shape the intended (and unintended) outcomes. While the reasons for intervention failure are often attributed to evaluation phase issues (eg, drop-out, underpowering or lack of adherence), the quality of the initial development process is fundamental to anticipating and averting later evaluation and implementation issues.^2^ Considering which development methods to use, and why, is therefore an essential part of reducing research waste,^3^ yet evidence for how development methods contribute to successful outcomes remains limited. For example, findings on whether using co-design methods improves outcomes are inconsistent,^4^ as is evidence on the effectiveness of behavioural theory-based approaches.^5^

Understanding the contribution of methodology to intervention success requires close attention to the development process itself. Despite its importance, this has been described as a ‘black box’, with decisions made about design and content often going under-reported.^6^ The INDEX study^7^ advanced understanding in this area by summarising the various development approaches used in the field,^8^ using an expert Delphi panel to define key principles^9^ and conducting a qualitative study to consider what contributes to perceived success according to researchers.^10^ Steps were also taken towards ensuring greater reporting transparency with the publication of the Template for Intervention Description and Replication checklist (TIDieR)^11^ and the GUIDance for the rEporting of intervention Development checklist (GUIDED).^12^

This prior work has focused on the field of intervention development in general, and less is known about how specific methodologies are applied in practice. Given the wide range of development frameworks available, there is a need to examine the use of individual approaches to help researchers select the appropriate methodology for their own study contexts. The Person-Based Approach (PBA)^13^ has been increasingly cited in the literature as a development method over the last decade,^14^ but it is currently unknown how it is applied in practice.

### The Person-Based Approach

The PBA was developed in response to concerns about the inefficiency of the established development and evaluation cycle. At the time, qualitative studies with users were almost exclusively conducted as process evaluations, which meant that issues of acceptability and feasibility were often only identified after costly trialling. The PBA was conceived to embed research with users early-on, and throughout the development process, so that content could be refined before evaluation. This was especially relevant in the context of the rapid growth in eHealth interventions, for which the issue of low user engagement has been a particular problem.^15^

Since the publication of the original methodology papers in 2015,^13^ the PBA has been widely adopted for developing interventions of various formats, including in-person, digital and hybrid programmes.^14^ Despite its growing adoption by study teams to develop interventions across these diverse settings, little is known about how and why researchers are choosing this approach, how they select which research activities to undertake or how these choices influence the research process, resource allocation or study outcomes.

The PBA is characterised by a collection of activities that aim to facilitate in-depth knowledge of those who will be affected by the intervention, and ways of accommodating those perspectives in intervention development (figure 1). It shares features with several well-established development and design methodologies,^8^ but is distinct in important ways. Although similar to user-experience (UX) and human-centred design (HCD) in a focus on understanding the views of the end-user, UX and HCD typically focus on enhancing engagement with a product (such as an app or website), whereas the PBA is primarily focused on achieving sustainable, offline behaviour change. Participatory design approaches are related and compatible with the PBA, but the PBA recommends combining co-design with qualitative research and iterative testing across a wide range of users. The aim is to ensure the development process incorporates the views of a broader target population than the co-design partners (who are often highly motivated advocates), including the views of potential users who may have more concerns about the intervention.

**Figure 1:**
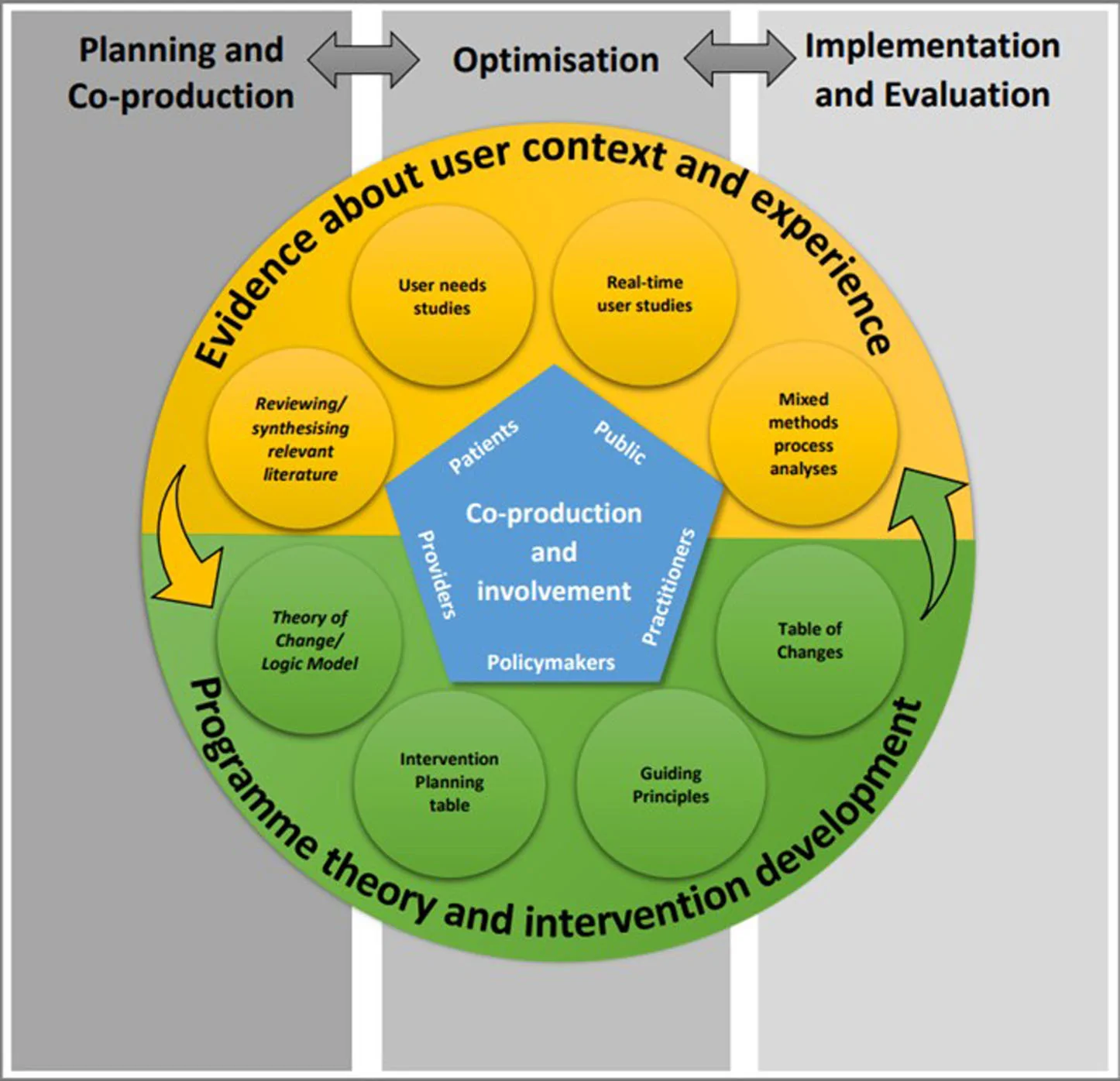
Overview of the Person-Based Approach, taken from https://personbasedapproach.org/pba_diagram.html, with permission of the site owners.

The PBA does not mandate specific methods to gather evidence; these can be selected and adapted based on context, resources and intervention aims. The PBA is distinctive in using structured tools for translating this evidence into content; for further details, see the descriptions of Guiding Principles and Table of Changes (ToC) in online supplemental table 1.

Central to the PBA is the early and ongoing involvement of stakeholders (we use ‘stakeholder’ throughout for consistency with the participants’ own terminology, but note the ongoing discussion on the use of this term and the proposed change to ‘interest-holders’),^16^ such as patients, care providers, clinical practitioners and policy makers. These groups can then be engaged with throughout the research timeline, varying from study oversight roles to participatory work.^17^ The PBA emphasises the value of involving intervention target users and stakeholders as co-producers of the intervention, rather than only as consultees however, to keep development grounded in the needs and realities of the people it is meant to serve (blue central area in figure 1).

In order to generate evidence about user context and experience (yellow area of figure 1), researchers may build on prior research and conduct literature reviews to identify evidence such as existing intervention effectiveness or known barriers to engagement. User needs studies to understand the target population are also key at this stage, particularly if there is a paucity of prior relevant evidence, and primary research can be collected to understand target-user perspectives and characteristics. Evidence about user experience of the intervention can also be generated as content is developed, and real-time user studies such as Think-Aloud (TA) interviews are used to optimise content, capture immediate reactions to prototypes and provide detailed feedback on the accessibility and acceptability of intervention materials.^18^ Rather than treating development as a linear process, the PBA uses continuous and iterative evaluation and does not just aim for one endpoint assessment. In this way, user-based evidence from qualitative and mixed-methods process analysis^19^ can be used to update previous development plans as well as the intervention content itself.^20^

The PBA supports the process of content development with tools that link the evidence on user needs with intervention programme theory, in order to specify the design decisions taken by the study team (green area of figure 1). The Guiding Principles^21^ articulate; (1) key user characteristics affecting engagement, (2) design objectives to overcome barriers and improve engagement and (3) the intervention features to achieve these objectives. Behaviour change theories and techniques can also be used across the whole PBA timeline, such as through the use of explanatory programme theory, through logic modelling or via behavioural analysis/mapping against proposed content.^22^ An Intervention Planning Table (IPT)^23^ can also be used to capture and link theory with evidence from user needs studies by including (1) descriptions of what specific intervention content needs to be included, (2) how the different sources of knowledge and evidence have informed the content decisions and (3) behavioural theory mapped to the proposed intervention features.

Once content has been developed, iterative cycles of testing with users can identify areas for improvement before it is ready for evaluation, and a ToC^24^ can be used to systematically prioritise the feedback from optimisation activities, by coding each insight for importance, feasibility and frequency. Typically, the Must do, Should do, Could do and Would like to do framework (MoSCoW)^25^ is applied to determine which modifications are essential versus those which might be desirable, depending on the available study resources. The evidence from Think-Aloud interviews is often the main source of this kind of optimisation feedback.

### Purpose of the current study

The intervention development process needs to be ‘dynamic, iterative, creative, open to change and forward looking to future evaluation and implementation’^9^ and the range of decisions research teams must make about which theories, methods and data collection tools to use are therefore considerable and significantly impact study timelines and resources, regardless of which development framework is used.

For the PBA, this challenge is compounded by its inherent epistemological openness, as it is deliberately non-prescriptive about methods, and researchers use their own judgement about what is most appropriate for their study contexts. How researchers make these decisions in practice has not yet been examined, and answering this question requires direct inquiry with people who have experience of using the approach.

While previous methodological work on intervention development has been central to characterising the range of approaches used across the field^8^ and establishing reporting guidelines,^12^ qualitative accounts of the process itself are rare. One prior qualitative study with intervention developers^26^ found that the design process (generating ideas and deciding on content, format and delivery) is difficult to articulate and disseminate within current publishing norms. What researchers actually do, and why, therefore remains under-documented in the literature. Given that the PBA has been increasingly cited as a development approach over the past decade, it offers a useful case for examining these issues. The current study therefore builds on the prior work on intervention methodology by asking why researchers chose the PBA, how they applied it under real-world constraints and what they perceived its strengths and limitations to be.

This kind of knowledge is usually shared informally within research collaborations and networks, and systematically exploring and disseminating it are important for two main reasons. First, it can offer insights for researchers considering using the PBA, by making visible the choices made under real-world constraints, and documenting what researchers actually do and why, and providing rare practice-based accounts of an intervention method. Exploring the strengths and challenges of the approach could also indicate areas for future improvement of the PBA, responsive to researcher needs. Second, this paper provides empirical accounts which can serve as the foundation for future evaluative work, as the contribution of researcher choice and use of methods to intervention success cannot be assessed without this being documented. More broadly, this paper can contribute to efforts in the field to open up the ‘black box’ of intervention development. While reporting standards such as TiDieR and GUIDED have improved the transparency of what development choices are taken, qualitative accounts can surface the reasons behind them.

## Methods

### Positionality and qualitative approach

This qualitative study has been reported in accordance with the Standards for Reporting Qualitative Research guidelines.^27^ A critical realist ontological stance has been taken, which views observable events as actualisations of unobservable mechanisms.^28^ This position allowed us to analyse the participants’ subjective experiences of the PBA and their professional praxis, while acknowledging the contextual factors affecting their perspectives, such as wider sociocultural influences on academic identities, research and dissemination.

The data from semistructured interviews were analysed using thematic methods based on Interpretive Description,^29^ a method designed specifically for health researchers looking for practice-relevant insights in their data, and a framework matrix^30^ was used to organise and synthesise the data. This combination is compatible with our critical realist stance, as it allows for the analytical consideration of both individual agency and perspectives, as well as broader, structural influences.

The primary researcher (LH) is a doctoral student with a background in behavioural science and prior experience with using the PBA. The study team are experts in the use of the PBA (SD) and included an originating author of the approach (LY).

### Participant sampling

Potential participants were identified through studies included in a scoping review on the use of the PBA for intervention development between 2015 and 2023.^14^ This strategy was an efficient way to identify potential participants. The authors of the originating PBA methods papers^13 31^ were excluded from the current study, in order to minimise bias.

Authors from the scoping study with publications post 2020 were eligible for invitation, and the most recent papers were prioritised for sampling to facilitate easier recollection of the studies. Due to the often-lengthy timelines of intervention research, however, some of the studies discussed in the interviews had commenced years before publication.

The contributorship statements in the papers were used to select which author(s) would be invited (eg, prioritising those responsible for methodology and undertaking primary research on the study). Snowball sampling was also used, with invited participants suggesting other potential interviewees (eg, a relevant co-author on a paper). In total, 105 direct invites were sent to 83 UK-based researchers and 22 non-UK. Response rates were comparable for both groups, and the predominance of UK-based researchers in the final sample therefore reflects the eligible and invited pool of potential participants.

We contacted participants by direct invitation through email, aiming to purposively sample for heterogeneity in geographical location and career stages. This sampling strategy was designed to capture a range of perspectives and experiences with intervention research. The recruited sample included 16 researchers from various disciplinary backgrounds (including psychology, medicine, allied healthcare and nursing), from PhD through to professorial stages of career. Most participants had backgrounds in developing multiple interventions for a range of settings and modes of delivery (eg, digital, hybrid, public health, primary care), reflecting the heterogeneity in the use of the PBA and the diverse experience of the interviewees.

Participants were mainly based in UK universities, with one participant based in Europe and one in North America (see online supplemental table 2 for characteristics). Although we did not succeed in recruiting participants from the Global South (GS), some studies under discussion in this paper were set in GS locations. However, the final sample reflects the current use of the PBA, as to date it has primarily been used by researchers based in the UK and in NHS settings.^14^

We had an initial target of ~20 participants for this study and ended recruitment with a final sample size of 16, using the ‘information power’ framework, which proposes sampling should be assessed against the contribution of knowledge from the analysis.^32^ Malterud *et al*^32^ describe that when participants are able to engage reflexively and articulately with the subject, this increases the informational yield per interview, reducing the number of participants needed. The interviews yielded accounts with high levels of critical thought and reflexivity on the part of the participants, from a purposive and ‘dense’ sample of experts on a defined and bounded topic. Additionally, both the researcher and the researched share a ‘disciplinary lens’,^33^ which facilitated open dialogue and enabled a contextualised analysis. Data were iteratively assessed for depth of contribution, and the collected data answer the informational needs of the study.

### Patient and public involvement

None.

### Data collection

All interviews were conducted by the first author (LH), via Microsoft Teams, between August 2024 and January 2025, and lasted between 35 min and 1 hour. These were guided by a flexible topic guide which focused on the participants’ experiences with using the PBA, such as their reasons for selecting specific methods, any challenges they encountered, any adaptations they made to the approach and their perception of the impact of the PBA on the overall intervention development process. The topic guide was updated iteratively as the interviews progressed, so the focus was on the intended research questions, while allowing for any new concepts raised by the participants (see online supplemental appendix).

### Data analysis

The interviews were recorded and transcribed using Microsoft Teams, before being edited for clarity in Microsoft Word, and uploaded to NVivo V.14 for analysis.

The thematic analysis was guided by Interpretive Description^34^ and moved through iterative stages of data familiarisation: the inductive and deductive coding of text segments into meaningful units, data synthesis and the development of overarching themes.

The deductive coding was drawn from the core methodological elements and phases of the PBA itself (see figure 1), such as TA interviews, Guiding Principles and the ToC, and these provided an initial coding structure. Inductive coding was conducted alongside this, capturing experiences beyond the structural conceptualisation of the PBA.

A framework matrix was also used as an organisational tool to facilitate data synthesis and to consider comparisons across participant data. Analysis was undertaken by the first author (LH), with a proportion of the coding and theming reviewed for consistency by the second author (SD).

A draft of the final paper was sent to the participants, to gain feedback on the contextualisation and anonymisation of quotes, and to share the results of the thematic analysis, checking if this was representative of their experience.

## Results

The participants in this study told us that using the PBA to guide the development of interventions provided them with a structured, yet adaptable framework that aided in the synthesis of different evidence sources, while keeping the focus on the end-user. Data were themed under three headings: *Navigation*, *Flexibility* and *Integration*.

### Navigation

This theme describes the way the participants used the PBA to guide themselves and project partners through the challenges and ongoing decisions of the development process. It includes the following subthemes: *A route map from ideas to content*, *Navigating digital development* and *Guidance through development*.

#### A route map from ideas to content

The participants in this study valued the PBA for providing structured guidance through development phases, providing a clear roadmap for progression that aided them in planning, carrying out and reporting research, and in creating content. Participants valued PBA methodology papers and used these as guides to move from what they conceptualised the needs of their population to be, towards actual intervention content and features.

How do we get from that idea to a fully worked up intervention? At that point … I thought I need, you know, some kind of approach to this. And that’s when the Person-Based Approach came in. (ID 1)

Participants often heard about the PBA through their professional networks, which enhanced its perceived legitimacy and credibility as a methodological approach. It also provided a recognised methodology that could be presented to external stakeholders, such as in grant applications, where the PBA was used to “detail your framework quite clearly” (ID 4) to potential funders. When planning their research, the participants told us it helped them select appropriate data collection activities at each stage. This methodological roadmap was used in different research scenarios, both for interventions starting from scratch with no prior evidence and for studies aiming to optimise or adapt existing interventions. The choice to use the PBA was consistently linked to the perspective that “it is useful in thinking about a framework for really involving people right from the beginning, getting it right” (ID 10), and participants told us they chose the PBA as part of an inherent commitment to prioritising user perspectives from the start of their research.

In terms of specific research tools, much of the PBA is drawn from already established research practices; however, participants consistently identified the ToC and the Guiding Principles as methodological elements that defined the approach as different or unique, and saw these as navigational aids for moving from ideas towards content. Both the ToC and Guiding Principles are tools designed to aid the challenges of progressing from conceptualising and understanding user needs to the creation of specific intervention features. For that reason, they were valued not only for guiding the research activities but also for documenting and presenting the direction of the research itself.

[the ToC] is a really good way of presenting evidence because you have a specific issue, a quotation from a participant, and then what you’ve brought, what you’ve understood from that quotation. So when you’re presenting it to funders or presenting it to even the participant, patient groups, it’s a really clear way of saying, “OK, well, this is what we found and this is the changes that we’ve made based on it”. So yeah, it’s really effective. (ID 2)

#### Navigating digital development

For the interview participants who had used the PBA to develop digital interventions, it offered an accessible way to engage with the design process, particularly for teams that typically had disciplinary backgrounds in healthcare or psychology. While they created initial prototypes themselves, they often had no formal expertise in this area, and the PBA was seen as useful for bridging the skills-gap between health research methods and digital design. One participant who did have formal expertise in user-experience (UX) design described a methodological alignment between the PBA and UX design principles:

When I’m teaching user-experience design, I do refer to the Person-Based Approach as an example of good user-experience design methodology. So, user-experience design is about understanding the user, and making sure any solution that you develop for them digitally is addressing a problem that they have, rather the problem that you believe they have, and that solves something…. accomplishes a goal for them in the real world. (ID 2)

Although participants described using the PBA as useful to navigate the practical steps needed to create and refine digital or visual content with users, for some there were still concerns that a lack of UX skills affected engagement.

I sometimes query what you’re testing, when you know, we have this real issue with up-take and engagement, and I think now that’s why people are thinking about, you know, we need to get in people that are specialists within those areas. (ID 15)

One of the key elements of the PBA is that it guides the research team through successive points of engagement with the target user as content is refined iteratively. This aligns with commercial approaches to UX design; however, participants working with external design teams were under pressure to minimise the number of prototype iterations that they could undertake to refine the content.

… we couldn’t test 10 different versions, because we couldn’t ask our design company to create 10 different versions because it was very expensive… We were mindful of the financial implication of that. (ID 12)

Keeping external partners on-track with the aims of the study in this way was described as essential. Clear communication with technology and design teams was not always successful, and there were accounts of failure in working relationships.

…after I’d given them the design brief I was just, hang on a sec, they’ve obviously gone away and just thought, well, we’ll just design our own thing. (ID 1)

Some participants used their ToC or their Guiding Principles to communicate their design brief to partners. This approach was taken to try and convey the importance of the underlying behavioural theory, and validate this with data from engagement with target users.

#### Guidance through development phases

The PBA provided participants with a structure to progress through intervention development phases, and participants valued how this guidance enabled them to evaluate their progress at key time points, and steer the overall direction of the study.

There was a progression of elements, so that was helpful to keep on track, and so things like having the Guiding Principles which I found just really useful, because it does help me focus at the different stages, and where you’ve got to, and whether it’s still relevant, and keeps you from going off on various tangents. (ID 6)

Participants told us they found the PBA particularly valuable during earlier development stages of planning and optimisation, describing it as “incredibly helpful for the planning stage” (ID 13), and of continued utility through content iterations. It was seen as filling a gap in existing development frameworks, and participants appreciated having a clear protocol for moving from initial conceptualisation to evidence gathering and integration, before prototype development. However, participants voiced uncertainty about how applicable the PBA was to later evaluation or implementation phases.

The PBA has helped us to get the building blocks that we can now really go and build on going forward. (ID 9)

My understanding of the PBA is that it certainly could be used throughout the process, but it probably has been used more so in [earlier] development because it’s about that kind of Person-Based Approach to developing interventions, and not necessarily to evaluating them as far as I know. (ID 3)

### Flexibility

The participants in this study described how using the PBA had helped legitimise flexibility in their choice of study activities, enabling them to better adapt to study contexts and resources, and that this flexibility was perceived as core to maintaining user-centred principles. This includes the following subthemes: *Resource responsiveness* and *Adaptations for study and user contexts*.

#### Resource responsiveness

Participants particularly valued the PBA’s flexibility when having to account for constraints on their study resources (such as time, funding and study expertise). They made pragmatic choices about which research activities to undertake, enabling them to preserve the integrity of their methods, while working within their resource limitations. This involved identifying what they believed to be the core values of the PBA, using them to guide overall practice, and then selecting and adapting research activities to suit the study context.

I tend to be quite a pragmatic person…. somebody who kind of takes the bits that make sense or that work. Not necessarily a person who will just adopt the whole approach and make sure we’re doing everything, you know, to the tee. Now my guess, my understanding of the PBA is that probably designed that way. You know, it’s to be quite flexible. (ID 10)

In particular, the flexibility of the PBA in integrating qualitative and/or patient and public involvement (PPI) work meant they felt able to maintain the PBA’s focus on understanding user perspectives while accommodating any practical limitations of the study resources.

So in that situation, the in-depth interview study at the beginning was dropped. We didn’t have time for that, and we just relied on two things. There’s already a lot of good qualitative research already out there about the experience of this particular group, we’re looking at the literature. And secondly, we have a really good strong PPI group who are very involved, very engaged, very knowledgeable and so, you know, those two things meant we still felt we had an idea of the user-perspective, and we weren’t going in completely blind. (ID 8)

#### Adaptations for study and user contexts

Participants had a dual understanding of TAs as generating immediate, actionable feedback for intervention refinement, while also collecting data for qualitative analysis. Participants sometimes prioritised practical design needs over qualitative insights, creating a more agile development process that still focused on their end-users, but enabled them to be responsive to study timelines.

I did analyse (Think-Alouds) properly for the write-up after, but actually the main analysis was that I had to get the feedback back to the app to the design team. (ID 1)

The participants in this study also adapted research approaches to better suit their target users and their cultural contexts to enhance the quality of responses. In a non-UK study setting, the researcher/participant dynamic of TAs was found to be a barrier to eliciting critical feedback, so researchers adapted to a different format while still accessing real-time thinking:

… rather than having solo Think-Aloud interviews we ran co-discovery sessions.^35^ That’s where you have two people looking at something together. Because then you have the conversation between those two people to observe rather than just a researcher questioning a participant. (ID 2)

For interventions with a large amount of digital content, participants described asking for TA feedback focused on certain sections, or particular elements. Where real-time user studies were impractical due to the mode/delivery of the intervention content (such as having no visual prompt material), reflexive interviews directly afterwards were used to try and access a similar kind of immediacy to TAs.

The format of the PPI activities was particularly varied; some saw this role filled by ‘expert patients’ and part of study oversight (eg, reviewing documentation), while others used PPI to reach out to target users in their own communities (eg, attending existing groups and events), or running participatory workshops (eg, to co-create Guiding Principles).

So the listening cafe was excellent for getting really honest feedback from a group of parents who would not want to talk to anyone wearing a lanyard, normally, so that was really, really helpful. (ID 5)

Participants consistently linked these kinds of adaptations to the flexibility they felt they had been given by using the PBA, and its focus on finding the best way to understand and connect with target users. Participants who went out to meet their users in their communities, and co-created study materials and ideas with them, described the PBA as giving them a means to undertake research that overcame barriers to research, such as hierarchy and formality, but could still meet the expectation of methodological rigour.

I think that definitely opened my mind to, yeah, more flexible ways of engaging people and making me think that that’s okay - it doesn’t have to be this very strict formulaic process that we all know, and therefore might fall back on. Instead, you know, we can think a bit more open mindedly and I can see then from my research that really worked as well. And it gives you more confidence to do that. (ID 13)

Participants in this study also described using different approaches to developing their Guiding Principles; some created them based on literature reviews and their own professional knowledge, others derived them from primary qualitative data and some developed them collaboratively with PPI groups. They also varied in how they used them throughout development, with some maintaining them as reference points, and others updating them based on new insights.

### Integration

The PBA was valued by participants in this study as a way to synthesise diverse evidence sources, including behavioural theory, prior evidence and the perspectives of users and stakeholders. They also felt able to combine the PBA with other methodological frameworks in order to select appropriate intervention development methods across the whole study timeline. This includes the following subthemes: *Balancing multiple sources of evidence*, *Integrating frameworks and theory*, *Integration of professional values* and *Integrative challenges*.

#### Balancing multiple sources of evidence

Participants in this study valued the PBA for providing a way to integrate multiple sources of evidence (such as behaviour change theory, evidence on the effectiveness of prior interventions and stakeholder expertise), while maintaining and centring target user perspectives. This process was not always easy, and they described the integration and weighting of differing evidence sources as requiring considerable judgement.

And so it’s kind of questioning that thing that, you know does… a user always know best? Do we always know best? So I guess trying to make those decisions. And so kind of incorporate that feedback in but again then you coming back at the end of day, coming back to the decision of you know, well you still need to kind of put that in the context of what we know from literature. What we know from previous research, what we know from our own experience of conducting these kinds of interventions. So then yeah, I guess it’s kind of putting that all together. (ID 10)

The PBA’s tools, particularly the ToC and Guiding Principles, were viewed as helpful in documenting this integration, by creating audit trails of how different evidence sources informed development decisions.

Well, the Guiding Principles were mostly from my lived experience and the [stakeholder groups]. It was meeting the two contexts, and what was the best fit for them, and trying to keep that in mind during the whole process. Is it flexible enough? Is it clear? We kind of bring all that back to those Guiding Principles during the whole project. (ID 11)

Participants often discussed the differences in undertaking PPI activities versus using qualitative research methods, particularly noting the differences in the people making up these groups, and how representative (or not) they were of the wider target population. Decisions on how to use PPI in a study were often informed by practical considerations around timelines, ethics approvals and finance, as well as the characteristics of the panels themselves.

I guess it’s pros and cons of different approaches… so we actually had PPI members who did the Think-Alouds for us … The advantage is it’s relatively rapid. You don’t have to go through ethics and all of that, but the disadvantage is our PPI group were very much not representative of the wider population. You know, they were very, very knowledgeable on the topic, they had very, very specific experiences that that might be quite unique and complex. (ID 8)

It was often challenging for studies with a diverse range of target users and stakeholders to prioritise feedback from these different groups, as well as with theory and evidence from prior research, but there was a consensus that understanding the target user was viewed as the foundation of PBA intervention work.

You mustn’t be afraid to diverge from theory if necessary, you know, they’re there supposedly to help us not to make us stick rigidly to them … that’s where I think the flexibility comes from, because it says you can and should be guided by the needs and views of your end user. (ID 1)

One participant suggested that the increasing use of participatory methods might disrupt established behavioural science paradigms and the top-down, theory-driven approach to development.

I think we really have to grapple with this idea, particularly now with the prevalence of co-production, almost feels like co-production could take out, a lot of the theory type stuff. But that could you know, that loses a lot of good evidence-base on how to develop interventions doesn’t it, so we need to work out how to get them to work together, and I think in doing that we need to reflect that the answer isn’t that they can only work together happily in one like nice, neat, tidy thing. It’s like we have to accept some of the messiness in it….and I think the PBA has helped us a long way doing that. (ID 4)

#### Integrating frameworks and theory

Participants viewed the PBA as complementary to other intervention development guides, particularly the Medical Research Council framework (MRC^36^). While the MRC was described as providing an overarching structure for the research process, participants valued the PBA for offering more detailed practical guidance:

[The MRC framework is a] guide for kind of you know where your research fits in and how you would kind of plan your studies, and what kinds of things you need to be doing. But then the PBA I think provides a bit more in terms of well, how do you actually go about it? And you know, how do we kind of put this into practice, and connect all the dots, and connect the stages, and feed it all in together. (ID 10)

The PBA was seen as meeting a gap in existing guidance by working within established development principles, and providing distinct, user-centred activities.

We followed the development section for [the MRC], especially because it goes through from development, feasibility, evaluation and implementation so that was the big influence and there was the, you know, the PBA in terms of having taken a user-centred approach. Having the co-production is important, really key. So it wasn’t just about gathering ideas at the start and then going on to develop something, but actually, having them, people involved throughout the development process, having a really iterative approach. (ID 7)

The integration of theory into development work was also a core feature of the study activities discussed by the participants, and this took diverse formats, depending on their views of the PBA’s role in this. One participant’s perception was that the PBA lacks specificity in offering behaviour change techniques (BCTs) to guide intervention features. They addressed this by using the Behaviour Change Wheel (BCW),^37^ which provides systematic guidance for selecting specific BCTs based on the identified needs of the user.

The limitation of the PBA is like, ‘OK, well, how do you then go from understanding the needs to actually what the specific content of the intervention is”? And sometimes the BCT types approach, so the Behaviour Change Wheel, that’s quite helpful there. (ID 4)

Another participant understood the PBA as primarily a tool to promote engagement with interventions, using it to optimise digital delivery of their content, and relying on BCTs and related frameworks (such as the Theoretical Domains Framework)^38^ for developing their core intervention mechanisms.

It was very theoretical, and the components included were theory driven, and evidence driven. And so the PBA was more used to determine *how* to deliver it, and what kind of add-ons to do, and in terms of more engagement-focused content, something like sending reminders … [When looking for theoretical-informed content] we have the literature … we looked at those which are common in interventions … there were pretty much two promising components came out of that, and they were the ones we added in (ID 16)

Another chose to integrate implementation theory early on, along with stakeholder involvement, believing that the PBA facilitated this approach:

… having a psychosocial model underlying the intervention is really, really important, so it felt like the Realist Evaluation could fit nicely with that, … and Normalisation Process Theory, I really still want to bring that in as early as possible because [the stakeholders] said “Don’t wait till implementation to get us in, get us in early”. And I know the PBA works really nicely with that. (ID 9)

Participants therefore used the PBA as part of a wider toolkit, rather than as a standalone methodology. They described combining the PBA with different frameworks and theories based on the perceived strengths and limitations of each approach, and how they could be carried out with the study resources available.

#### Integration of professional values

Beyond methodological considerations, participants described how the PBA enabled them to integrate their professional values into their research practice. Many were initially drawn to the approach because of its alignment with their existing commitment to user-centred research:

Instantly was drawn to… how person-centred it is. I really like the fact that it feels like it would make the work very relatable to real people, and I liked that and the fact that I’d get to do loads of kind of engagement and involvement work. (ID 5)

This value-based reason for choosing the PBA was a consistent pattern in the data, with participants orienting towards a research practice that prioritised end-user input throughout the development process. For these researchers, the PBA helped them to enact inclusive research principles that aligned with their core research values. Participants also described how the PBA aligned with their epistemological preferences, particularly those who favoured iterative approaches over more linear methodologies:

It is about understanding things qualitatively … you’ve got this health services research comes with quite a positivist view and “we want to follow something and we want to do it, and then it’ll be done, and it’ll be right”. But actually, that isn’t really what it is, and the (PBA) is saying actually it isn’t “Just do this, you do this and it’s right”. It’s like “you do this, and you understand this, and then you iterate this” - you’re trying to make it very iterative - building qualitative reflective understanding into a set of processes. (ID 4)

#### Integrative challenges

Despite stating that explanations and examples of the PBA in the literature were valued, publishing their own PBA work was described as challenging. Participants told us that there was sometimes limited space to describe the process of development in papers, often due to the target journals’ scope and criteria. The participants viewed this as part of a wider pattern in health research dissemination, where evaluation and results are typically prioritised.

I think authors should publish how they developed the intervention and not just focus on the big trial paper… I think it needs to be done in a quite structured and evidence-based approach … and not just say yeah, ‘tick’, we’ve talked to [the target user group]’… there needs to be some public and patient involvement. But I think that there’s still a lack of guidelines how to do that. (ID 7)

Beyond dissemination challenges, participants also described practical difficulties with core PBA components. Despite identifying Guiding Principles as a defining feature of the PBA, many found them conceptually difficult to understand and integrate into the process of development.

Maybe it’s not clear what they are in the old papers from 2015. But I look at them, I think I understand, but then when I write them, I don’t know… The students don’t understand them. I don’t know why it’s hard… I think there are examples, but maybe not clear guidelines, maybe like a broader definition or what Guiding Principles are with some examples. (ID 12)

## Discussion

### Summary of main findings

This study explored why researchers chose the PBA, how they applied it under real-world study constraints and what they perceived its strengths and limitations to be. A central finding was that researchers valued the PBA for providing structured guidance to plan and carry out development work within study resources (*Navigation*) and flexibly adapt methods to their participants and iterate/refine content (*Flexibility*); as well as integrate diverse evidence sources, behavioural theory and other development frameworks (*Integration*). A consistent finding was that researchers were drawn to the PBA because it aligned with the personal and professional value they placed on user-centred practice.

Surfacing this practice directly addresses the under-reporting of methodological decision-making and shares the practical knowledge more typically exchanged within smaller research networks, making it visible to the wider disciplinary community. This study reveals that a primary driver for methodological practice is meeting user needs over fidelity to strict procedures, which is then enacted through pragmatic and resource-responsive choices about methods. This is in alignment with an established argument in complex intervention science that what should be held constant is the underlying *function* or principle of an approach, while its specific *form* should adapt to context.^39^ This has direct consequences for the evaluation of interventions, however, and how the features which lead to successful outcomes can be identified and assessed, which we will turn to next.

### The value and evaluation of user involvement

There are high rates of research waste in intervention development and problems with ‘voltage drop’ from efficacy to effectiveness,^40^ with interventions often failing in real-world contexts.^41^ Participants in this study believed that using the PBA helped them create interventions that would work in the real-world due to sustained user involvement in development, which would identify and anticipate barriers that might otherwise emerge in implementation. The PBA shares these features with UX, HCD and participatory approaches (see the INDEX taxonomy);^8^ however, for researchers without a background in design or technology, it made the benefits of these methods accessible and offered a structured way to integrate participatory work with other evidence.

The PBA does not preclude the use of other frameworks, and participants also used other approaches, including the BCW and MRC guidance, to specify behaviour change content or plan for implementation. Evaluating the contribution of intervention methods to intervention success is therefore not straightforward. As this study confirms, development approaches are routinely combined rather than used in isolation, often with no uniform fidelity to a single framework. The MRC guidance^36^ advocates for diverse evaluative perspectives to account for complexity, including efficacy, effectiveness, theory-based and systems approaches, which vary in their offer of explanatory depth, comparability and feasibility.

Since no single, constant method can be isolated, explanatory accounts of why particular configurations of development activities contribute to success could be sought via theory-based approaches (eg, realist evaluation).^42^ The current study represents an early step towards such evaluation, by documenting the conditions under which researchers chose and adapted the PBA in practice, and provides an empirical basis from which to examine the hypothesis that embedding user involvement early and throughout development anticipates later barriers, reducing costly failures at evaluation and beyond.

### The implications of flexible methodological practice

The flexibility valued by participants raises issues around researcher accountability and ethical engagement with users. When research methods are used flexibly, the rigour of their application will substantially depend on the skill of the researcher.^29^ In the absence of a fixed or formal process, rigour is then evidenced by the transparent reporting of research activities.

The boundary between qualitative and PPI work also deserves some scrutiny. Using a PPI approach can be a more rapid and agile way to engage with users and does not require ethics approval under UK research governance, the regulatory framework within which the PBA was developed.^43^ However, co-production with PPI contributors involves ethically sensitive decisions, for example, concerning who is involved in a study and what roles and protections contributors have, as well as how study results can be analysed and shared.^17^ Again, reporting what approach was chosen, and what happened, supports rigour, ethics and research transparency.^27^

Checklists such as TIDieR^11^ support the description of final intervention content; however, reporting studies against GUIDED^12^ would also make the adaptive decisions described in this study more visible. Where PPI contributors have been involved in intervention co-production, this should also be reported using frameworks such as Guidance for Reporting Involvement of Patients and the Public (GRIPP2^44^) in order to make clear what the involvement consisted of, who contributed and how it shaped the intervention, rather than these decisions being justified as a response to resource pressures alone.

These kinds of decisions require balancing resources, researcher skill, ethical obligations, disciplinary norms and the needs of both the study and the end-users. Reporting them transparently allows that reasoning to be understood and appraised by others, but participants described having limited opportunities to describe these fully. While checklists can capture some of this, they cannot convey rationale, which requires dedicated space to describe intervention methods, as well as results.^45^

### Theory: Top-down and generative approaches

Traditional approaches to the use of theory in intervention development involve the top-down application of established behavioural theories. Such approaches use validated models and prior evidence to predict the behavioural influences and select appropriate BCTs, and in many cases, participants in this study used this approach. However, the PBA also enabled the integration of theory chosen during cycles of user engagement, rather than predetermined. Other participants iterated ideas about what would work based on user feedback and insights, without describing this as formal theorising. This differs from formal *theory application* and represents a form of *theory generation*, where user engagement helps develop a context-dependent, theoretically rich understanding of what will work for the target user and why.^46^ The PBA’s methodological openness appears to accommodate both these forms of theorising during the development process.

### Tailoring qualitative and PPI methods

Participants described tailoring their qualitative and PPI approaches to better suit their intervention contexts and target populations and tried to address power imbalances in researcher-participant dynamics^47^ by adapting their methods of data collection for real-time user feedback, or by making efforts to ‘de-formalise’ their engagement with communities to make this accessible. Such adaptations recognise that cultural differences and power dynamics can affect participant engagement in traditional research formats, and demonstrate that the PBA can be used as a methodological scaffold for more equitable practices.

### Refining the Person-Based Approach to meet researcher needs

Despite voicing predominantly positive experiences, participants identified areas where additional guidance or refinement could be valuable. The most frequently described was difficulty in understanding the remit and application of the Guiding Principles. Current training resources on these might need to be enhanced with worked examples of how they can be developed (eg, from literature, primary data and co-production with PPI groups), in order to ensure they can be used confidently by researchers.

While participants consistently valued the PBA during the planning and optimisation stages, they expressed uncertainty about its utility for later evaluation and implementation phases and frequently described using other frameworks and/or theories for these stages, such as Normalisation Process Theory,^48^ the MRC guidance or the integrated-Promoting Action on Research Implementation in Health Services framework (i-PARIHS).^49^ The PBA is therefore used alongside other evaluation and implementation science frameworks. There may be a need for clearer guidance on how the PBA can be best integrated into feasibility, evaluation and implementation phases, as well as its relationship to other phase-specific frameworks, to support researchers using the PBA as part of a toolkit, rather than a standalone approach.

### Limitations of the current study

Our sampling strategy may have introduced selection bias, as researchers with positive PBA experiences might be over-represented due to being more likely to respond to recruitment invitations. Participants might have also felt pressure to present experiences that aligned with professional expectations about doing things ‘the right way’, rather than discussions of taking ‘wrong-turns’ in their studies.

The framework matrix revealed no differences by career stage or professional background. This may reflect a sample too homogeneous in their view of the PBA to show them (perhaps due to the sampling issue described above), or that these characteristics are not influential. We found that researchers were drawn to the PBA due to their existing commitment to user-centred practice, and this motivation may cut across career stage and profession.

The sample was predominantly UK based, limiting the transferability of findings to other research cultures and healthcare systems. The participants’ methodological choices may therefore particularly reflect the expectations of UK funders such as the National Institute for Health and Care Research (NIHR), which has placed growing emphasis on the use of PPI in research.^50^ Since funding contexts significantly influence what development methods are prioritised and resourced, the experiences reported here may not be representative of how the PBA could be used in international settings.

Sample size was determined using an information power framework, and while 16 participants was considered sufficient due to the shared academic lens between researcher and participant which facilitated rich dialogue, a larger or more diverse sample may have surfaced experiences not represented here. Additionally, the proximity of the interviewer and authors to the origins of the PBA may have influenced the recruitment response, interview dynamics and the interpretation of findings.

## Conclusions

This study has provided practical accounts and perspectives of how researchers use the PBA for intervention development. A key finding is that meeting user needs requires adapting not only intervention content but also the research activities themselves. Using the PBA gave researchers confidence to adapt their qualitative and PPI methods, such as by going out to community settings, redesigning data collection to reduce power imbalances and co-creating Guiding Principles and intervention content with target users. This matters because health research must reach the communities who face the greatest barriers to traditional research participation. These findings suggest that the use of the PBA aligns with the epistemological and professional values of researchers committed to user-centred practice, and can support more inclusive approaches to intervention development. This reflects the broader disciplinary recognition that prioritising the participant experience in health research, particularly when conducted in partnership with diverse communities, requires moving beyond traditional, hierarchical approaches.^51 52^

Participants described combining the PBA with other frameworks and methods, rather than adhering to a single approach, confirming that evaluating the contribution of development methodologies to intervention success would be challenging.^7^ Without evidence of how, why and in what context development approaches are selected and applied, it will not be possible to consider their contribution to the studies’ research efficiency or cost-effectiveness. Future work in this area will therefore need to account for the complexity inherent in the development process, considering not simply which methodology was used, but how and why particular configurations of development activities lead to successful outcomes. Achieving this will mean paying attention to the choices of the researchers themselves, in order to understand the relationship between study contexts and study methodologies. If we want to understand why interventions work for the people they are designed to benefit, we will also need to understand the decisions made by the people that create them.

## Supplementary material

10.1136/bmjopen-2025-112780 online supplemental file 1

## Data Availability

No data are available.
