# Supplementary material for: Understanding research practices and methodological choices when applying the Person-Based Approach to health intervention development: A qualitative interview study
Source: BMJ Open. 2026 Oct 7;16(10):e112780. doi: 10.1136/bmjopen-2025-112780 (PMC13646946; doi:10.1136/bmjopen-2025-112780)
Supplement: Supplementary file 1 [file bmjopen-2025-112780.reviewer_comments.pdf]

PEER REVIEW HISTORY

BMJ Open publishes all reviews undertaken for accepted manuscripts. Reviewers are asked to complete a checklist review form and are provided with free text boxes to elaborate on their assessment. These free text comments are reproduced below.

ARTICLE DETAILS

Title (Provisional)

Understanding research practices and methodological choices when applying the Person-Based Approach to health intervention development: a qualitative interview study.

Authors

Holt, Lydia; Denford, Sarah; Yardley, Lucy

VERSION 1 - REVIEW

**Reviewer**	**1**
**Name**	**Balzer, Katrin**
**Affiliation**	**Universität zu Lübeck, Institute for Social Medicine and Epidemiology**
**Date**	**05-Dec-2025**
**COI**	**none**

Review on the manuscript bmj-open-2025-112780

Thank you very much for the opportunity to review this manuscript reporting on a qualitative study that investigated methodological motives and experiences with the application of the Person-Based Approach (PBA) in the intervention development.

The subject under study is relevant for the advancement of methodological frameworks and methods for the development of complex, predominantly non-pharmacological interventions in healthcare, including those working with digital components. Thus, theoretically, this study holds important scientific potential.

However, based on the current manuscript, the rationale for this study and the knowledge (to be) added by this work remain unclear. The results provide some insights into the purposes, likely advantages and challenges of the use of PBA, but no specific needs for further development of the PBA, e.g. with regard to conceptualization of certain research phases in the intervention development, epistemological or methodological principles or reporting requirements, were neither derived nor even discussed. This lag also includes

potential reflections on how to assess the benefits of certain methodological approaches, be it from an ethical or a clinical (outcome-driven) view.

Thus, overall, the scientific value of this manuscript appears too low to recommend consideration of publication. Below, please find a list of further major comments. If these comments will be addressed by the authors, the relevance and value of this study might become clearer. I wish the authors much success in their further work on this manuscript.

Major comments

(1) Background

*The subject of this study needs to be specified further. The types of interventions of interest that could be developed framed by the PBA should be clearly defined. Currently a mix of health behaviour change interventions in general (see e.g. first para of the introduction) and digital interventions is being addressed. That challenges the interpretation of the findings.

*The particular characteristics and instruments of the PBA should be described more distinctly. The PBA elements (Appendix Table 1) should be part of the main manuscript and briefly introduced in the background chapter. It should become transparent to less informed readers, how PBA can be applied in each single phase of intervention development. A figure visualizing the scope and methods of PBA for each phase would be useful.

*It should become clear, in which respects PBA differ from other methodological frameworks for intervention development and participatory research, e.g. human-centered design.

*The background chapter should end with a distinct description of the lack of knowledge regarding use of PBA that was addressed by this study. The authors state that their study may help reduce “research waste” (last para background chapter). However, this assumed consequence is not plausible to me. What was to be found out by the study – for which purpose?

(2) Methods

*Dependability and reflexivity of the study findings: The study authors report that they are experienced in the application of the PBA, with one author having been involved in the development of this methodological approach. These experiences probably largely framed the recruitment of the participants, data collection and analysis, and eventually the findings. Thus, the findings may be bound to this very specific study contexts A larger study team, also including further experts in intervention development and representatives of the patient-perspective, would add rigor to the findings. Additionally, limitations due to higher risk of dependability and less reflection by other involved parties should be discussed in the limitations.

*Participant sampling:

- What was the desired sample size, and how was it justified.

- Did the authors analyse a priori which research groups in the different regions worldwide had already used PBA? How were these groups spread geographically? And how many of them were invited to participate? (see also limitations)

*Data collection: To facilitate interpretation of the findings, it would be useful if one example of a topic guide is provided as supplement in the appendix.

*Data analysis:

- The authors report that they used an inductive-deductive approach for the coding of the data. What was the reference for the deductive part of the data analysis? What kind of preconceptions, theories or research findings informed the analysis?

- Did the authors use peer debriefing to strengthen the credibility of the findings?

-How did the authors assess data saturation?

(3) Results

*Unfortunately, this chapter reads quite lengthy. It contains several repetitions, as, for example, it refers to challenges in the use of the PBA in several sub-chapters.

*Generally, sub-categories/sub-themes of the central themes would be beneficial to the structure of the results. Such sub-categories/sub-themes would also help clarify the scope of this study and reduce repetitions across the several themes.

(4) Discussion and limitations

*In the discussion, the results should be more explicitly discussed against existing methodological research and discussions in the area of intervention development (in the relevant field of interest). For example, comparisons with reported experiences in the application of other intervention or technology development approaches appear very useful and would improve the scientific richness of this manuscript: What are important lessons learned and future prospects for the development/update of frameworks for intervention or technology development? What is already known about the impact of PBA or other relevant frameworks on the processes and outcomes of intervention/technology development?

*The limitations also need to be expanded. The authors should reflect on the data saturation of the findings, especially the unilateral UK-based focus (only 1 participant each from Scandinavia and North America), and the close relationship of at least two authors to the PBA.

(5) Conclusions

The conclusions need to be more focused, and precise as well.: What are the main findings and insights? And what is to do in the theory and research practice on intervention/technology development? Only results should be summarized here that have been reported earlier in the manuscript. For example: The last para addresses subjects that are not directly linked to the findings of the present study.

VERSION 1 - AUTHOR RESPONSE

Reviewer: 1

Comments to the Author:
Review on the manuscript bmj-open-2025-112780
Thank you very much for the opportunity to review this manuscript reporting on a qualitative study that investigated methodological motives and experiences with the application of the Person-Based Approach (PBA) in the intervention development.
The subject under study is relevant for the advancement of methodological frameworks and methods for the development of complex, predominantly non-pharmacological interventions in healthcare, including those working with digital components. Thus, theoretically, this study holds important scientific potential.
However, based on the current manuscript, the rationale for this study and the knowledge (to be) added by this work remain unclear. The results provide some insights into the purposes, likely advantages and challenges of the use of PBA, but no specific needs for further development of the PBA, e.g. with regard to conceptualization of certain research phases in the intervention development, epistemological or methodological principles or reporting requirements, were neither derived nor even discussed. This lag also includes potential reflections on how to assess the benefits of certain methodological approaches, be it from an ethical or a clinical (outcome-driven) view.
Thus, overall, the scientific value of this manuscript appears too low to recommend consideration of publication. Below, please find a list of further major comments. If these comments will be addressed by the authors, the relevance and value of this study might become clearer. I wish the authors much success in their further work on this manuscript.

Thank you for these helpful comments, we will endeavour to address these in our revision, and hope that they will strengthen the manuscript.

We agree that a discussion of the implications for the future development of the PBA is missing. Drawing on our findings, we have identified two main areas where additional guidance would be valuable: the conceptualisation and application of Guiding Principles, (which participants found difficult to operationalise), and clarity about the scope of the PBA across the full intervention development timeline. These are now highlighted in the section ‘Refining the PBA to meet researcher needs’.

We would note, however, that defining specific changes to the methodology falls outside the scope of what a qualitative interview study can appropriately deliver. Formal recommendations or mandated changes would be better developed by a consensus

exercise, such as a Delphi study. The contribution of the current study is instead to surface the experiences, perceptions, and practice-based insights of researchers who have used the PBA, which could provide a foundation for such a future consensus exercise.

With regards to *"This lag also includes potential reflections on how to assess the benefits of certain methodological approaches, be it from an ethical or a clinical (outcome-driven) view"* .

We agree that this is an important issue for intervention development science, and have expanded on this in the Discussion section in our revision (see also response to Major comments)

Major comments
(1) Background
*The subject of this study needs to be specified further. The types of interventions of interest that could be developed framed by the PBA should be clearly defined. Currently a mix of health behaviour change interventions in general (see e.g. first para of the introduction) and digital interventions is being addressed. That challenges the interpretation of the findings.

The PBA was originally developed in the context of digital health interventions, but has since been widely adopted across a broad range of intervention formats and health contexts, including in-person, digital, and hybrid programmes. This heterogeneity is therefore a defining characteristic of the PBA itself. We have clarified this in the Background, making explicit that the study intentionally encompasses this range, and that participants' experiences reflect this and have worked with diverse intervention settings (noted in the Sampling section).

*The particular characteristics and instruments of the PBA should be described more distinctly. The PBA elements (Appendix Table 1) should be part of the main manuscript and briefly introduced in the background chapter. It should become transparent to less informed readers, how PBA can be applied in each single phase of intervention development. A figure visualizing the scope and methods of PBA for each phase would be useful.

*It should become clear, in which respects PBA differ from other methodological frameworks for intervention development and participatory research, e.g. human-centered design.

We have added a figure visualising the PBA across development phases, however both this and the PBA elements table originate from previously published sources, so we

have left the table in the Appendix to avoid over-use of previously published material in the main paper. We have also added a paragraph to the Background distinguishing the PBA from related methodologies

*The background chapter should end with a distinct description of the lack of knowledge regarding use of PBA that was addressed by this study. The authors state that their study may help reduce “research waste” (last para background chapter). However, this assumed consequence is not plausible to me. What was to be found out by the study – for which purpose?

Thank-you for this important observation.

The study set out to answer questions that methodology papers and literature reviews cannot address; why researchers choose the PBA; how they apply it under real-world constraints; and what they perceive its strengths and limitations to be. The purpose was to generate practice-based evidence that is currently absent from the literature. To clarify this, we have substantially revised the Background.

We have also refined our consideration of research waste throughout the manuscript, particularly in the Discussion section, and clarify that we do not expect this paper to directly reduce research waste, but that it contributes to a small but growing literature on how intervention development methodologies are used in practice. Such evidence is needed to support researchers making methodological decisions which impact significantly on the allocation of study resources. The PBA was originally created in response to concerns that user views were only sought during or after trialling, rather than as part of the development process, and therefore the method itself directly engages with the topic of research waste. This has been clarified in the Background (‘The Person-Based Approach’ section).

Exploratory qualitative evidence will also be needed before any theoretical and evaluative framework could be designed to assess methodological contributions to research efficiency. We propose in the Discussion that a realist evaluation could address this issue, and consider the kinds of cost-effectiveness-specific programme theories that could be drawn from the findings of the present study.

(2) Methods

*Dependability and reflexivity of the study findings: The study authors report that they are experienced in the application of the PBA, with one author having been involved in the development of this methodological approach. These experiences probably largely framed the recruitment of the participants, data collection and analysis, and eventually the findings. Thus, the findings may be bound to this very specific study contexts A larger study team, also including further experts in intervention development and representatives of the patient-perspective, would add rigor to the findings. Additionally,

limitations due to higher risk of dependability and less reflection by other involved parties should be discussed in the limitations.

We agree that the relationship of the authors with the PBA is a legitimate consideration, and we have now addressed issue this more fully in the Limitations section. However, established methodological frameworks are not static, for example the MRC guidance for developing and evaluating complex interventions has undergone revisions. The PBA should continue be subject to iterative, evidence-based methodological refinement, those with proximity to the methods should be involved, and qualitative inquiry will be an important part of this process.

The following reflexivity safeguards were in place: participants were assured that Professor Yardley would have no access to transcripts or know participant identities; independent coding review was undertaken by a second author (SD); and member-checking with all participants was conducted on the final themes.

*Participant sampling:
- What was the desired sample size, and how was it justified.
- Did the authors analyse a priori which research groups in the different regions worldwide had already used PBA? How were these groups spread geographically? And how many of them were invited to participate? (see also limitations)

We have added a section on our sampling, and how we considered information power on the section 'participant sampling'. We explain how we used an existing scoping review to identify authors who had published PBA papers since 2020. The review confirmed that the majority of researchers using the PBA are UK based and undertaking research in NHS settings.

*Data collection: To facilitate interpretation of the findings, it would be useful if one example of a topic guide is provided as supplement in the appendix.

We have added this to the supplemental material

*Data analysis:
- The authors report that they used an inductive-deductive approach for the coding of the data. What was the reference for the deductive part of the data analysis? What kind of preconceptions, theories or research findings informed the analysis?

We thank the reviewer for identifying this omission. The deductive coding frame was drawn from the core methodological elements and phases of the PBA itself (as described in the Background and illustrated in Figure 1), including tools such as Think-Aloud interviews, Guiding Principles, and the Table of Changes. These provided the

initial coding structure. Inductive coding ran concurrently, remaining open to experiences that went beyond the structural conceptualisation of the PBA. This has been added to the Data Analysis section.

- Did the authors use peer debriefing to strengthen the credibility of the findings?

A draft manuscript was sent to participants so they could assess how their contributions were used in context, and to sense check the overall theming. (see Data Analysis section)

-How did the authors assess data saturation?

We used information power, please see the expanded Participant Sampling section

(3) Results
*Unfortunately, this chapter reads quite lengthy. It contains several repetitions, as, for example, it refers to challenges in the use of the PBA in several sub-chapters.
*Generally, sub-categories/sub-themes of the central themes would be beneficial to the structure of the results. Such sub-categories/sub-themes would also help clarify the scope of this study and reduce repetitions across the several themes.

We have addressed this by ensuring participants' perceived limitations are within the themes to which they most relates, rather than in the previous standalone 'Challenges' section.

We have made the Results structure more explicit by adding an introductory sentence at the start of each theme that lists the sub-themes to follow, so readers can better navigate the understand the scope of each theme before reading it.

Navigation: (i) A route map from ideas to content; (ii) Navigating digital development; (iii) Guidance through development phases.

Flexibility: (i) Resource responsiveness; (ii) Adaptations for study and user context.

Integration: (i) Balancing multiple sources of evidence; (ii) Integrating frameworks and theory; (iii) Integration of professional values. (iv) Integrative challenges

(4) Discussion and limitations
*In the discussion, the results should be more explicitly discussed against existing methodological research and discussions in the area of intervention development (in the relevant field of interest). For example, comparisons with reported experiences in the application of other intervention or technology development approaches appear very useful and would improve the scientific richness of this manuscript: What are

important lessons learned and future prospects for the development/update of frameworks for intervention or technology development? What is already known about the impact of PBA or other relevant frameworks on the processes and outcomes of intervention/technology development?

We agree that the Discussion would benefit from more explicit situating of findings in the wider literature, although comparable methodological research in intervention development is currently limited. We have nonetheless strengthened the Discussion's engagement with the existing literature by positioning the study in relation to the INDEX study, and the GUIDED reporting guidelines.

We have also deepened the section on user involvement and the potential impact on research waste. We acknowledge that testing whether user-centred development reduces research waste is genuinely difficult using conventional economic evaluation because, as this study confirms, development approaches are routinely combined rather than used in isolation, and used adaptively, not prescriptively. We therefore use the concept of Realist evaluation as a way to address this issue, illustrated with examples drawn from our findings.

*The limitations also need to be expanded. The authors should reflect on the data saturation of the findings, especially the unilateral UK-based focus (only 1 participant each from Scandinavia and North America), and the close relationship of at least two authors to the PBA.

We have significantly revised the Limitations section. As well as the section on the proximity of the authors to the approach, we have addressed the predominantly UK-based sample and the implications for transferability, the consideration of information power and sample size.

(5) Conclusions

The conclusions need to be more focused, and precise as well. What are the main findings and insights? And what is to do in the theory and research practice on intervention/technology development? Only results should be summarized here that have been reported earlier in the manuscript. For example: The last para addresses subjects that are not directly linked to the findings of the present study.

We agree that the original final paragraph of the Conclusions drew on broader disciplinary literature in a way that was not sufficiently grounded in the findings of the study. The broad point about moving beyond hierarchical research approaches has been kept, but we now position this as following on from the findings of the current study, and the conclusion now stays closer to our data.

VERSION 2 - REVIEW

**Reviewer**	**1**
**Name**	**Balzer, Katrin**
**Affiliation**	**Universität zu Lübeck, Institute for Social Medicine and Epidemiology**
**Date**	**12-May-2026**
**COI**	

bmjopen-2025-112780.R1

Thank you very much for the opportunity to review the revised manuscript reporting on a qualitative study that shed light on the rationales for and experiences with using the Person-Based Approach (PBA) in intervention development.

The authors substantively adapted some parts of the manuscript, especially the background, methods and discussion sections, in response to the reviewers' comments. With this revision, most of my earlier comments have been well addressed. There are only some remaining or newly introduced issues that should be reconsidered to strengthen the completeness of the report and the scientific value of the findings. I wish the authors much success in their further work on this manuscript.

Major comments

(1) My most substantial concern still relates to the rationale for this study. Although the authors revised the background section substantively and added a specific research question, I still wonder what the scientific importance of this study and the findings is. Why it s useful to have deeper insights in researchers motivations to use the PBA approach and the experiences strengths and challenges? The purpose – i.e. not the aim – of this study remains still hidden. How are the results assumed to matter to future research and researchers? This should be made even clearer, both in the background section and later in the discussion.

(2) Among the strength and limitations (study highlights), the fourth bullet point stresses the fact that the data were analysed by means of matrix in order to identify potential cross-participant patterns but no such differences were identified. They question the usefulness of this analytical approach. However, one might also conclude that these lacking patterns point to limitations related to the sample. Perhaps, the participants were too uniform in their positive attitudes towards PBA to contain sufficient variance to reveal any typical differences. Also, one cannot rule out that the researched characteristics career level and professional background simply do not have an important role for the researchers' view on PBA. To my understanding, the authors' argument falls short.

(3) Furthermore, it has to be noted, that the results section does not include any finding that indicates application of the framework matrix and the lacking patterns/differences. Reported results contain only a few quotes embedded in the main text. These quotes are too few, and the narrative summary of the findings on the subcategories does not give any insights into comparisons or contrasting analyses across the participants. Therefore, the discussion of the limited usefulness of the matric approach come slightly “out of the blue” in the limitations section.

(4) Background, section “The Person-Based Approach”: The description of key features of the PBA largely circles around the iterative PBA-framed intervention development cycle displayed in figures 1. However, I found it sometimes quite difficult to connect the information given in the text to the content of the figure. The text suggests a certain sequence of phases or steps in intervention development that are displayed as chronically ordered steps in the figure. Also, some steps such as “moving into design” are not included in the figure at all. To improve the clarity of the description, the text should be more congruent to the logical structure of figure 1.

(5) Methods, participant sampling:

*It should also be reported how many researchers/authors (representing which countries) were invited to participate in this qualitative study.

*Do the authors have assumption about potential reasons for the dominance of UK-based researchers in the sample?

*Also, the authors should clarify in the text whether a certain sample size had been considered in advance or not. The newly added paragraph about the sample size justification suggests that these considerations were made post-hoc. And I hesitate to agree with the authors’ view that the sample of this study has been purposively recruited and shows high specificity. As already argued above in my comment on the lacking findings from the framework matrix, I am concerned that the sample might have been too homogeneous in their views on PBA and criteria that may influence these views, e.g. kind of previous research experiences with qualitative and quantitative methods and experiences with PPI.

*Table 2, participant characteristics: Information about the types of interventions that were subjects of the participants’ PBA experience would be useful (e.g. digital intervention, patient education etc.).

(6) Discussion

*Though the authors revised the first para, the current version still lacks a concise summary of the main findings. First the main findings should be summarized before their relevance will be stressed. For most parts, the first paragraph would suit better to the justification of the study in the background section.

*The value and evaluation of user involvement:

(i) This paragraph emphasizes the specific value of PBA due to very early and consistent involvement and participation of/co-creation with representatives of the target groups. However, I question that this is an unique attribute of PBA. Also other development frameworks such as human-centered design claim this feature as well, and there are many studies that implement participation and co-creation very consequently but do not or would not title their design as PBA. Thus, this section would benefit if the key features highlighted by the interviewees would be discussed against other established methods, leading to the requirements for robust and feasible meta research on the relative merits and challenges of the single frameworks.

*The authors discuss potential evaluation approaches, with stressing potential advantages of use of Realist Evaluation, e.g. due to questionable feasibility of control conditions. However, this exclusive focus on realist approaches appears a bit narrow and eclectic, since there are also other evaluation approaches typically addressing complex interventions (such as research approaches), both at primary (e.g. mixed methods studies) and secondary research levels (e.g. mixed methods reviews). For a scientific discussion of evaluation options, a more nuanced, less single-sided discussion of pros and cons of various evaluation approaches would be advantageous.

Minor comments

(7) Language and formal issues

There are still some incomplete sentences or other typos in the text, e.g.

* Background, section "The Person-Based Approach", second to last paragraph: The second sentence appears incomplete, probably it should be as "… has not been examined, and …"

*Participant sampling, second to last paragraph: In the second sentence, the word "number" is mentioned twice in the row.

*Discussion, "The value and evaluation of user involvement": Why are parts of the last paragraph set in italic letters?

VERSION 2 - AUTHOR RESPONSE

Reviewer: 1
Prof. Katrin Balzer, Universität zu Lübeck
Comments to the Author:
bmjopen-2025-112780.R1
Thank you very much for the opportunity to review the revised manuscript reporting on a qualitative study that shed light on the rationales for and experiences with using the Person-Based Approach (PBA) in intervention development.

The authors substantively adapted some parts of the manuscript, especially the background, methods and discussion sections, in response to the reviewers'

comments. With this revision, most of my earlier comments have been well addressed. There are only some remaining or newly introduced issues that should be reconsidered to strengthen the completeness of the report and the scientific value of the findings. I wish the authors much success in their further work on this manuscript.

Major comments

(1) My most substantial concern still relates to the rationale for this study. Although the authors revised the background section substantively and added a specific research question, I still wonder what the scientific importance of this study and the findings is. Why it s useful to have deeper insights in researchers motivations to use the PBA approach and the experiences strengths and challenges? The purpose – i.e. not the aim – of this study remains still hidden. How are the results assumed to matter to future research and researchers? This should be made even clearer, both in the background section and later in the discussion.

We thank the reviewer for pressing us to clarify the purpose over the aim of the study, and the opportunity to make this clearer. We have added a section at the end of the Background, giving the 2 main reasons as 1) formally investigating and disseminating practice-based knowledge which is under-reported and more usually shared within smaller networks, and providing other researchers with information with which to choose their own methods amidst the growing field of intervention development frameworks; and 2) providing a foundation for future evaluation of intervention methods, as their influence on successful outcomes cannot be assessed without empirical accounts.

We have also restructured and added to the first part of the Discussion section to reflect this.

(2) Among the strength and limitations (study highlights), the fourth bullet point stresses the fact that the data were analysed by means of matrix in order to identify potential cross-participant patterns but no such differences were identified. They question the usefulness of this analytical approach. However, one might also conclude that these lacking patterns point to limitations related to the sample. Perhaps, the participants were too uniform in their positive attitudes towards PBA to contain sufficient variance to reveal any typical differences. Also, one cannot rule out that the researched characteristics career level and professional background simply do not have an important role for the researchers' view on PBA. To my understanding, the authors' argument falls short.

We apologise, as our original point was not sufficiently detailed, we were not attempting to argue that the method is not useful, nor invalid across our analysis, but that our analysis did not yield the comparative data we sought on any demographic 'case'

variance. As the reviewer suggests, this uniformity could reflect a sample that was too homogeneous in its positive views to reveal such differences. However, this finding also aligns with a theme from our results, that the participants had a shared positionality, in their commitment to user-centred practice, and so we have expended on this to explain that this may cut across career stage and discipline (hence the lack of difference in the matrix). We have therefore integrated this into the Limitations section in the main manuscript.

(3) Furthermore, it has to be noted, that the results section does not include any finding that indicates application of the framework matrix and the lacking patterns/differences. Reported results contain only a few quotes embedded in the main text. These quotes are too few, and the narrative summary of the findings on the subcategories does not give any insights into comparisons or contrasting analyses across the participants. Therefore, the discussion of the limited usefulness of the matric approach come slightly “out of the blue” in the limitations section.

To clarify the concern around the presentation of the results; the analysis, interpretation and presentation of the results are in line with Interpretive Description, the thematic approach we used. Within this, we also used a matrix as a tool to summarise the data, due the many elements used within the PBA (e.g. Guiding Principles, Table of Changes, PPI , interviews, Think Alouds etc). However, we did not undertake a full Framework Analysis, which as the Reviewer notes, would indeed require a more systematic comparison or contrasting analyses across the participants. We have now clarified this in the Methods section.

We would like to emphasise that the analysis in fact did reveal great variation in the sample, for instance in the ways the Guiding Principles were created, how PPI was conducted, how theory was or was not integrated, and these are all evidenced in the Results and the quotes used. Variation and the major theme *Flexibility* are central findings of this paper, so we do not feel that the sample was too homogenous to investigate the central topic of this paper, that of methodological practice. However we present this as Interpretive Description, rather than systematic comparison.

As our analysis was Interpretive Description, our use of a matrix was primarily organisational, to summarise the data, and NVivo requires a characteristic to be entered as a case attribute (such as role or career level), to create the comparison matrix. There was variation in the subject of inquiry (methodological practice), but this was not found to be patterned according to the case attributes (e.g. participant characteristics). We think this is likely due to the participants shared commitment to user-centred practice cutting across these differences, and we have stated this in the paper.

(4) Background, section “The Person-Based Approach”: The description of key features

of the PBA largely circles around the iterative PBA-framed intervention development cycle displayed in figures 1. However, I found it sometimes quite difficult to connect the information given in the text to the content of the figure. The text suggests a certain sequence of phases or steps in intervention development that are displayed as chronically ordered steps in the figure. Also, some steps such as “moving into design” are not included in the figure at all. To improve the clarity of the description, the text should be more congruent to the logical structure of figure 1.

Thank you for the opportunity to update this section, we agree this would be an improvement to clarity. The manuscript has now been edited to group the narrative description of the PBA into the grouped sections of Figure 1, and these are referred to directly in the text (as blue PPI core, yellow evidence generation methods, and green theory and intervention development tools). We hope this improves the descriptive alignment between the text and the figure.

(5) Methods, participant sampling:
*It should also be reported how many researchers/authors (representing which countries) were invited to participate in this qualitative study.
*Do the authors have assumption about potential reasons for the dominance of UK-based researchers in the sample?
*Also, the authors should clarify in the text whether a certain sample size had been considered in advance or not. The newly added paragraph about the sample size justification suggests that these considerations were made post-hoc. And I hesitate to agree with the authors’ view that the sample of this study has been purposively recruited and shows high specificity. As already argued above in my comment on the lacking findings from the framework matrix, I am concerned that the sample might have been too homogeneous in their views on PBA and criteria that may influence these views, e.g. kind of previous research experiences with qualitative and quantitative methods and experiences with PPI.

Apologies for missing out this information, this has now been added to the manuscript in Sampling section ad we give further information below.

In total, 105 researchers were invited to participate, 83 based in the UK and 22 elsewhere, of whom 16 took part. We do not report by country to protect participant anonymity, given the small and potentially identifiable PBA researchers outside the UK. The UK predominance in the sample mirrors that of the eligible and invited pool, and response rates were similar for UK and non-UK invitees.

We attribute the UK dominance in the sample to the majority of eligible participants being UK based, as evidenced in the scoping review cited in the paper (Holt et. al 2025).

This in itself might be due to the approach spreading via institutional collaborations in UK, and professional networks (although we have not stated this in the manuscript as we have not direct evidence for this). Additionally, UK funders (particularly the NIHR) emphasise the importance of PPI and user-centred methods, which is what the PBA was designed for. We address these points in the Participant Sampling and Limitations section, as well as the Strengths and Limitations Box-out points. It is also a purpose of this paper to share practice based knowledge on the PBA beyond existing research networks

RE: Response rates, although eligibility included authors publishing from 2020 onwards, the PBA is often applied early, and intervention research has long timelines. By the time of the interviews in 2025, those at the earlier end of this window would have been trying to recall work potentially undertaken several years before their 2020 publication, and may have declined because of this. We believe the final sample was therefore skewed toward a smaller number of authors of more recent studies, for whom recall was more feasible.

We have also clarified the sample size in the manuscript. A target of ~ 20 participants was made in our ethics application. During recruitment, data adequacy was then assessed against the information power framework. To address the concern around specificity, in Malterud's framework, this is how dense and relevant the sample is to the study aim, not how internally varied it is. Our sample is very specific in this sense, as all participants were researchers discussing their own studies in-depth. In terms of information power, participants varied a great deal in how they used qualitative and PPI methods, and in how they used theory, (reported in *Flexibility* and *Integration* themes), but they shared a commitment to user-centred practice (our *Integration of professional values* sub-theme). We believe this latter is why we found no differences by career stage or discipline.

We accept the sample was largely positive about the PBA, and we describe reasons for this in our Limitations. We don't believe however, that a neutral sample was not really available here, as researchers who do not value the PBA are unlikely to invest in using it, so any sample will tend to be positively orientated. Consistent with our purposive design and use of information power, we sampled for rich, in-depth accounts from those with direct experience. Our aim was to share why and how researchers chose and used the PBA. Their decision to invest in the approach is what introduces a positive skew, but also what makes our participants informative.

*Table 2, participant characteristics: Information about the types of interventions that were subjects of the participants' PBA experience would be useful (e.g. digital intervention, patient education etc.).

We recruited participants through a specific paper, but the researchers often had broader PBA experience, as well as with other intervention frameworks. Importantly, most had worked on interventions of different types, settings, and modes of delivery, so tabulating the paper by which we contacted them would risk misrepresenting the breadth of experience each participant could draw on, and therefore would not reflect how the data were generated. Instead, we describe the range of intervention types across the sample in the text, rather than linking a single type to each participant.

(6) Discussion
*Though the authors revised the first para, the current version still lacks a concise summary of the main findings. First the main findings should be summarized before their relevance will be stressed. For most parts, the first paragraph would suit better to the justification of the study in the background section.

A summary of the main findings has been added to the first paragraph of the Discussion, and refence to prior studies moved to the Background.

*The value and evaluation of user involvement:
(i) This paragraph emphasizes the specific value of PBA due to very early and consistent involvement and participation of/co-creation with representatives of the target groups. However, I question that this is an unique attribute of PBA. Also other development frameworks such as human-centered design claim this feature as well, and there are many studies that implement participation and co-creation very consequently but do not or would not title their design as PBA. Thus, this section would benefit if the key features highlighted by the interviewees would be discussed against other established methods, leading to the requirements for robust and feasible meta research on the relative merits and challenges of the single frameworks.
*The authors discuss potential evaluation approaches, with stressing potential advantages of use of Realist Evaluation, e.g. due to questionable feasibility of control conditions. However, this exclusive focus on realist approaches appears a bit narrow and eclectic, since there are also other evaluation approaches typically addressing complex interventions (such as research approaches), both at primary (e.g. mixed methods studies) and secondary research levels (e.g. mixed methods reviews). For a scientific discussion of evaluation options, a more nuanced, less single-sided discussion of pros and cons of various evaluation approaches would be advantageous.

We do not claim that the PBA is unique in including methodological features also found in UX or participatory work (see text highlighted in yellow in the Background in thr marked copy), and we are cautious about responding to this comment, as it is not our intention to set-up a comparison between the PBA and other frameworks. We particularly want to avoid a framing that would argue for the merits of one methodology over another.

However, we do agree that some clarity on the overlap between the PBA and other methods would be helpful, and added text on this, as well as referring readers to an existing work, the INDEX study, which systemically compares intervention approaches. We also emphasise that the PBA can be, and is, used complementary to other development approaches.

Our leaning on Realist methods was an attempt to propose a potential answer to the vexed question of evaluation, however, this was doing both too much and too little so we have softened this, removing the worked example, as this reached beyond the scope of our study. We have also placed our proposal for Realist methods alongside the plurality of evaluation methods recommended by the MRC.

Minor comments
(7) Language and formal issues
There are still some incomplete sentences or other typos in the text, e.g.
* Background, section "The Person-Based Approach", second to last paragraph: The second sentence appears incomplete, probably it should be as "... has not been examined, and ..."
*Participant sampling, second to last paragraph: In the second sentence, the word "number" is mentioned twice in the row.
Thank-you, these errors have now been rectified

*Discussion, "The value and evaluation of user involvement": Why are parts of the last paragraph set in italic letters?

Across the paper we have used italics to stress important concepts e.g. names of themes, and to highlight comparative concepts e.g. the difference roles of *form* and *function* in intervention content, and the difference in *theory application* and *theory generation*. We will remove these if they are not conventions used in the journal

---

VERSION 3 - REVIEW

**Reviewer**	**1**
**Name**	**Balzer, Katrin**
**Affiliation**	**Universität zu Lübeck, Institute for Social Medicine and Epidemiology**
**Date**	**12-Jul-2026**
**COI**	

Review on manuscript bmjopen-2025-112780.R2

Thank you very much for the opportunity to review the once again revised version of this manuscript exploring researchers' experiences with and views on the use of the Person-Based Approach (PBA) in complex intervention research. Most of my previous comments have been sufficiently addressed so that the manuscript now shows more clarity and plausibility.

There are only a few (minor with one exception) critical points left that should be addressed before the manuscript will be considered for publication (please see below). I wish the authors much success in their final work on this insightful study report.

*Study purpose, newly added paragraph "The kind of information presented in this paper …": This sentence sounds as if it already goes to anticipate the results, what would not be appropriate for the description of the study aims. Also, my understanding is that the results of this study go beyond mere "information" but provide systematically explored insights into the researchers' conceptual and methodological knowledge. Therefore, I would like to suggest that the authors consider rewriting this sentence, e.g. as follows: This kind of knowledge is usually shared informally …, and systematically exploring and disseminating is important …".

*Discussion: I appreciate the revision of the summary of findings; it now provides a completer and more accurate account of the findings. However, to my perception, the successive discussion of the key findings still reads slightly too much framed by an uncritical reflection of the interview results. For example, while the PBA's flexibility, that was particularly valued by the interviewees, implies practical advantages in the complex intervention development process and allows for multifaceted openness, these subjectively perceived merits also raise questions regarding the reproducibility and validity of the intervention research process and touches research ethics. For a research paper, I would expect that the authors' discussion contains a well-balanced reflection on potential chances and threats associated with detected results of the study, i.e. in this case enriching and challenging implications of the detected rationales and practices for/in using the PBA for the strength of the intervention development process. For example, in my view the finding that advantages of the PBA were seen to help circumventing ethical approval (see Results/Balancing multiple sources of evidence), would require some critical reflection. Also, a brief critical reflection of potential implications for the reporting of PBA-based intervention development might be useful. That would both contribute to a more balanced tone of the discussion and emphasize the scientific relevance and potential impact of the authors' findings.

*Formal issues:

- Use of the term "stakeholders": Although I have not brought up this issue in my previous comments, I would like to raise the authors' awareness to the currently ongoing discussion about the negative connotation of this term and the recommended use of the term "interest-holders" instead (Akl et al. 2024, https://doi.org/10.1002/cesm.70007).

- Use of the abbreviation PBA: Although this abbreviation when the term “Person-Based Approach” is mentioned for the first time in the main text, it is not consequently used throughout the successive chapters. There are several instances where the full term is used instead. I recommend strict use of the abbreviation after its explanation at the first mentioning in the Background chapter.

- Discussion, newly introduced para “Surfacing this praxis directly …”: Is the term “praxis” correctly used here? While the English language is not my mother tongue, this term appears quite unfamiliar to me.

VERSION 3 - AUTHOR RESPONSE

Reviewer: 1
Prof. Katrin Balzer, Universität zu Lübeck
Comments to the Author:
Review on manuscript bmjopen-2025-112780.R2

Thank you very much for the opportunity to review the once again revised version of this manuscript exploring researchers’ experiences with and views on the use of the Person-Based Approach (PBA) in complex intervention research. Most of my previous comments have been sufficiently addressed so that the manuscript now shows more clarity and plausibility.
There are only a few (minor with one exception) critical points left that should be addressed before the manuscript will be considered for publication (please see below).
I wish the authors much success in their final work on this insightful study report.

Response: We are very grateful to Prof. Balzer for her thorough and detailed engagement with our manuscript over 3 rounds. Her feedback has improved the overall clarity and enabled a critical review that has strengthened the paper. Thank you very much for the time and attention paid.

*Study purpose, newly added paragraph “The kind of information presented in this paper …”: This sentence sounds as if it already goes to anticipate the results, what would not be appropriate for the description of the study aims. Also, my understanding is that the results of this study go beyond mere “information” but provide systematically explored insights into the researchers’ conceptual and methodological knowledge. Therefore, I would like to suggest that the authors consider rewriting this sentence, e.g. as follows: This kind of knowledge is usually shared informally …, and systematically exploring and disseminating is important …”.

Response: We have now updated the sentence so as not to anticipate the results.

*Discussion: I appreciate the revision of the summary of findings; it now provides a

completer and more accurate account of the findings. However, to my perception, the successive discussion of the key findings still reads slightly too much framed by an uncritical reflection of the interview results. For example, while the PBA's flexibility, that was particularly valued by the interviewees, implies practical advantages in the complex intervention development process and allows for multifaceted openness, these subjectively perceived merits also raise questions regarding the reproducibility and validity of the intervention research process and touches research ethics. For a research paper, I would expect that the authors' discussion contains a well-balanced reflection on potential chances and threats associated with detected results of the study, i.e. in this case enriching and challenging implications of the detected rationales and practices for/in using the PBA for the strength of the intervention development process. For example, in my view the finding that advantages of the PBA were seen to help circumventing ethical approval (see Results/Balancing multiple sources of evidence), would require some critical reflection. Also, a brief critical reflection of potential implications for the reporting of PBA-based intervention development might be useful. That would both contribute to a more balanced tone of the discussion and emphasize the scientific relevance and potential impact of the authors' findings.

Response: We thank the reviewer for this important point, and have added s new subsection to address this : "The implications of flexible methodological practice."

This reflects on the implications of the flexibility participants valued: that rigour depends on researcher skill and is therefore evidenced by transparent description; that PPI work, while more rapid and not requiring ethics approval under UK research governance, involves ethically sensitive decisions, and that reporting guidance (GUIDED, GRIPP2) has a role in making these choices visible

*Formal issues:
- Use of the term "stakeholders": Although I have not brought up this issue in my previous comments, I would like to raise the authors' awareness to the currently ongoing discussion about the negative connotation of this term and the recommended use of the term "interest-holders" instead (Akl et al. 2024, https://doi.org/10.1002/cesm.70007).

Response: We thank the reviewer for raising this, though aware of this discussion, the PBA itself and the studies discussed by the participants were undertaken and published before the paper on terminology, therefore we retained this for consistency with the results section and prior PBA papers. However, we have added a footnote to this effect to direct readers to the paper on the use of the term as we agree it should be highlighted.

- Use of the abbreviation PBA: Although this abbreviation when the term "Person-Based

Approach" is mentioned for the first time in the main text, it is not consequently used throughout the successive chapters. There are several instances where the full term is used instead. I recommend strict use of the abbreviation after its explanation at the first mentioning in the Background chapter.

Response: Thank-you, we have made this update

- Discussion, newly introduced para "Surfacing this praxis directly ...": Is the term "praxis" correctly used here? While the English language is not my mother tongue, this term appears quite unfamiliar to me.

Response: We have retained "praxis", where it offers a way to articulate how professional knowledge is enacted, (rather than merely held), but changed to 'practice' or similar where this does not affect our intended meaning of the text.
