# Supplementary material for: Understanding research practices and methodological choices when applying the Person-Based Approach to health intervention development: A qualitative interview study
Source: BMJ Open. 2026 Oct 7;16(10):e112780. doi: 10.1136/bmjopen-2025-112780 (PMC13646946; doi:10.1136/bmjopen-2025-112780)
Supplement: Supplementary file 2 [file bmjopen-16-10-s001.docx]

# Appendix

## Appendix Table 1: PBA elements

Reproduced from Holt L, Denford S, Bowers H, et al. The person-based approach to intervention development: A scoping review of methods and applications. DIGITAL HEALTH. 2025;11. doi:10.1177/20552076241305934. Licensed under CC BY 4.0.

| **PBA element / activity** | **Methods can include, but are not limited to:** |
| --- | --- |
| **Literature review** | Used flexibly to suit the needs of the project e.g. can use systematic, scoping, or qualitative/mixed methods synthesis to search for evidence on the effectiveness of interventions, potential content, barriers and facilitators to engagement, or relevant behaviour change theory. |
| **User Needs Studies** | Qualitative research such as interviews, focus-groups, ethnography.  Population level data analysis to identify specific characteristics. |
| **Intervention Planning table** | Captures and summarises information from research activities, PPI and stakeholder engagement, linking this to proposed intervention content and relevant theoretical frameworks of behaviour change. |
| **Guiding Principles** | These guide intervention development by specifying: 1) the key user characteristics which affect engagement, 2) a few key design objectives to improve engagement, and 3) features of the intervention which could meet the design objectives. |
| **Real-time user studies / Think-Aloud interviews** | A form of qualitative interviewing whereby users engage with an intervention and are prompted to describe their experiences to the researcher in real-time. Provides very detailed feedback on accessibility and acceptability of intervention materials. |
| **Table of Changes** | Tabulates feedback from activities designed to evaluate and optimise intervention content (e.g. Think-Aloud, process analysis, PPI/stakeholder discussion). Provides a coding framework to guide decision making when agreeing changes to an intervention. |
| **Patient and Public / Stakeholder engagement** | The involvement of those who the intervention could affect, such as potential users and potential providers. This is very variable depending on the project, for example, including oversight of all research processes, participatory workshops to create content, or giving rapid feedback during optimisation. Multiple PPI approaches are recommended to maximize diversity and ensure accessibility. |
| **Behaviour change theory** | Behaviour change theory can be incorporated into the PBA via:   - Programme theory, explaining how the intervention is meant to achieve its goals. - A logic model, summarising how the key elements of the intervention should lead to intended outcomes. - The guiding principles, describing how the design supports engagement. - The Intervention planning table and/or the table of changes, recording why intervention elements were chosen or amended. - A behavioural analysis, which links theory to the desired behaviour change, the relevant content and the intended outcomes. |
| **Process analysis** | Provides qualitative, quantitative or mixed-methods data on the how the users experience the intervention in a real-life context, and over a period of time. |
| **Co-design / Participatory research** | Defined as “meaningful end-user engagement in research design”^1^ these approaches take many forms and can employ a variety of methods. |

## Appendix Table 2: Participant characteristics

| **Characteristic** | **Category** | **Number of participants** |
| --- | --- | --- |
|  |  |  |
| **Country** | North America | 1 |
|  | Scandinavia | 1 |
|  | UK | 14 |
|  |  |  |
| **Discipline** |  |  |
|  | Human Computer Interaction | 1 |
|  | Medicine | 2 |
|  | Allied Health Professional | 2 |
|  | Nursing | 2 |
|  | Psychology / Behavioural Science | 9 |
|  |  |  |
| **Years of Experience** | 0-5 years | 3 |
|  | 5-10 years | 2 |
|  | 10-15 years | 5 |
|  | 15+ years | 6 |
|  |  |  |
| **Role** | PhD candidate | 3 |
|  | Academic Clinical Fellow / Senior Academic Clinical Fellow | 2 |
|  | Lecturer / Senior Lecturer | 2 |
|  | Allied Health Professional | 1 |
|  | Research Associate / Senior Research Associate | 3 |
|  | Research Fellow / Senior Research Fellow | 3 |
|  | Reader | 1 |
|  | Professor | 1 |

## Topic Guide:

NB: As studies vary considerably in methods used, not all questions will be relevant, and will be adapted for each interviewee.

**(Introductions, opportunity for questions and confirmation of consent)**

**Background**

Can you describe your professional background and experience in research?

**Definitions**

Why did you choose the PBA (for the paper/study under discussion)?

What sources of information or guidance did you rely on for making this choice (previous literature, webinars etc)?

What do you think are the core elements of the PBA?

**Design, development and content**

Did you start with novel or use existing content? How and when was this decided?

Can you tell me what phases of ID you used the PBA across? Why, and what other approaches did you use if other?

Can you describe any work you undertook partnering with external tech/design teams?

**Evidence**

What methods did you use to collect primary data and why? Can you describe how the context of your intervention (e.g., target population, setting) influenced this choice?

If conducted Think-alouds, can you describe how you conducted these?

**Person, PPI/Stakeholder**

What did your work with stakeholders and PPI groups look like ?

How was this integrated with other evidence from the study?

What did you do in the case of contrasting views?

**Theory**

If you created Guiding Principles, how were these developed?

Can you describe any use of behaviour change theories?

**Other**

How did you prioritise different type of knowledge when deciding on content?

Can you tell me how you disseminated your development work?

**Closing**

Would you/have you used the PBA again, or different ID approaches?

Any benefit / challenges in using PBA which led to this decision

What aspects of the PBA would you like to see covered in a future studies/literature/training

## Appendix References

1.	Slattery P, Saeri AK and Bragge P. Research co-design in health: a rapid overview of reviews. *Health Research Policy and Systems* 2020; 18: 17. DOI: 10.1186/s12961-020-0528-9.
